# Evaluating the Hydrological Components Contributions to Terrestrial Water Storage Changes in Inner Mongolia with Multiple Datasets

**DOI:** 10.3390/s23146452

**Published:** 2023-07-17

**Authors:** Yi Guo, Naichen Xing, Fuping Gan, Baikun Yan, Juan Bai

**Affiliations:** 1 China Aero Geophysical Survey and Remote Sensing Center for Natural Resources, China Geological Survey, Beijing 100083, China; guoyi92@163.com (Y.G.);; 2 Key Laboratory of Aerial Geophysics and Remote Sensing Geology, Ministry of Natural Resources, Beijing 100083, China

**Keywords:** TWS, lake water, soil moisture, GWS, GRACE, Inner Mongolia

## Abstract

In this study, multiple remote sensing data were used to quantitatively evaluate the contributions of surface water, soil moisture and groundwater to terrestrial water storage (TWS) changes in five groundwater resources zones of Inner Mongolia (GW_I, GW_II, GW_III, GW_IV and GW_V), China. The results showed that TWS increased at the rate of 2.14 mm/a for GW_I, while it decreased at the rate of 4.62 mm/a, 5.89 mm/a, 2.79 mm/a and 2.62 mm/a for GW_II, GW_III, GW_IV and GW_V during 2003–2021. Inner Mongolia experienced a widespread soil moisture increase with the rate of 4.17 mm/a, 2.13 mm/a, 1.20 mm/a, 0.25 mm/a and 1.36 mm/a for the five regions, respectively. Significant decreases were detected for regional groundwater storage (GWS) with the rate of 2.21 mm/a, 6.76 mm/a, 6.87 mm/a, 3.01 mm/a, and 4.14 mm/a, respectively. Soil moisture was the major contributor to TWS changes in GW_I, which accounted 58% of the total TWS changes. Groundwater was the greatest contributor to TWS changes in other four regions, especially GWS changes, which accounted for 76% TWS changes in GW_IV. In addition, this study found that the role of surface water was notable for calculating regional GWS changes.

## 1. Introduction

Global warming accelerates the water cycle processes and directly impacts the spatial and temporal distribution of water resources [1,2,3,4,5]. In addition, the intensive human activities exacerbated the uncertainty of water resources changes [6,7]. Under the pressures of climate changes and anthropogenic activities, arid regions being faced with complex water resource problems, as ecosystems in such regions are usually fragile and limited by water availability [8,9,10,11]. The question of how water resources in arid regions change under the dual influence of climate changes and increasing human activities was a topic of great concern and debate [12,13].

Inner Mongolia Autonomous Region (Inner Mongolia), China, is a typical arid and semiarid region with regional water shortage due to scarce precipitation. Furthermore, water resources overexploitation caused by human activities aggravated the local water shortage. Water resources shortage is the most important factor that restricts the social development, ecological security and agricultural production in this region. Previous studies preliminarily explored the water resources statues [14,15,16,17,18] and conducted the water resources supply and demand balance analyses [19,20,21]. The large regional span of Inner Mongolia makes the significant spatial differences in hydrological conditions. However, few studies revealed the spatial-temporal variations of water resources in Inner Mongolia, due to the lack of in situ monitoring data.

The advances in satellite remote sensing provide a valuable tool for monitoring water resources changes [22,23,24,25], such as precipitation [26,27], evaporation [28,29], lakes water [30,31], soil moisture [32,33,34], groundwater [35,36] and total terrestrial water storage (TWS) [37,38]. Guo et al. [39] evaluated the temporal and spatial changes of TWS in Inner Mongolia during 2003–2021 based on Gravity Recovery and Climate Experiment (GRACE) and GRACE-Follow on (GFO), which revealed the major driving factors. However, challenges still remain in studying the water resource changes in Inner Mongolia.

One challenge is how different hydrological components contribute to the total TWS changes. TWS is defined as all forms of water stored above and underneath the surface of the Earth [40,41]. GRACE and GFO proved to be reliable for monitoring TWS change at large spatial scales [42,43,44,45]. As GRACE/GFO is not able to measure vertical profile of TWS changes, auxiliary data are used to isolate TWS changes into individual hydrological component [46], such as evapotranspiration [47,48], basin discharge [49,50], soil moisture [51,52] and groundwater storage (GWS) [53,54,55]. Machine learning techniques are employed to extract valuable insights from the large volumes of remote sensing multiply data that are generated for mapping terrestrial water changes [56] and monitoring areas with high hydrological changes [57] and might be used for evaluate component contributions as well. Revealing the contributions of different storage components to total TWS changes is crucial for understanding the water cycle processes and water resources management. However, a critical evaluation of the contributions from individual hydrological component to TWS changes is yet to be conducted in Inner Mongolia.

In addition, it is difficult to quantitatively reveal the responses of water resource changes to natural climate changes and anthropogenic activities. For a given region, the TWS changes observed by GRACE/GFO are the comprehensive responses to both climate changes and human activities. Therefore, TWS changes based on GRACE/GFO have certain limitations in distinguishing the natural and human-induced changes [12]. Supplementary data are required to partition the total TWS changes into the natural and human-induced changes. For example, the human-induced TWS changes could be estimated by the differences between total TWS changes based on GRACE/GFO and natural TWS changes which estimate by meteorological factors.

The large regional span of Inner Mongolia makes the significant spatial differences in hydrological conditions. Therefore, in this study, Inner Mongolia was first divided into different subregions according to hydrological conditions. Then, GRACE/GFO data were used to examine the spatial and temporal behavior of water storage in Inner Mongolia. Additional remote sensing data, including satellite images and land surface models, were compiled to study the changes in water cycle elements (precipitation, evapotranspiration, surface water and soil moisture). The objectives of this study were to provide the comprehensive view of the contribution of different hydrological components to total water resources changes in arid regions, and comprehensively decompose the water resources changes caused by climate changes and human activities with the help of multi remote sensing observations.

## 2. Data and Methods

### 2.1. Study Area

**Figure 1:**
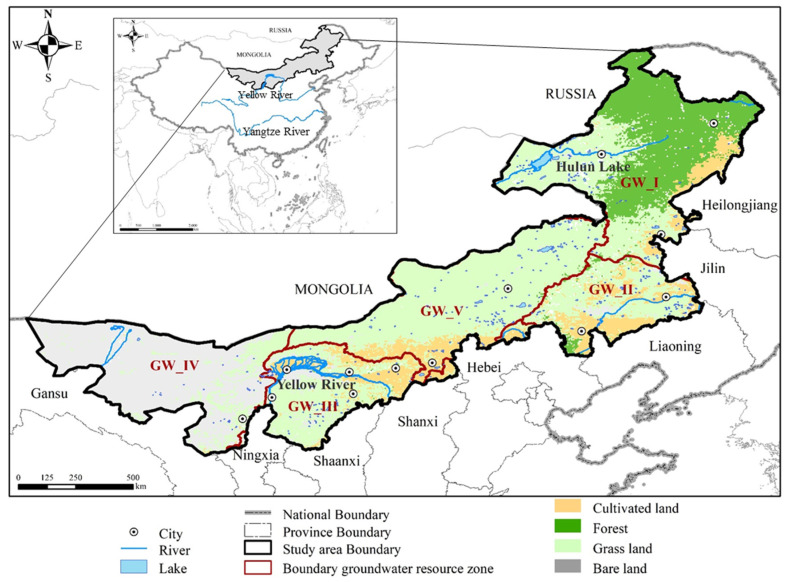
Location and the land use of Inner Mongolia.

Inner Mongolia Autonomous Region (referred to as “Inner Mongolia”) is located in the northern frontier of China, stretching diagonally from northeast to southwest in a narrow and long shape. The linear distance is more than 2400 km in east–west from 97°12′ to 126°4′, and 1700 km in north–south from 37°24′ to 53°23′. The study area covers a total area of 1.183 million square kilometers, accounting for 12.3% of China’s land area. Inner Mongolia is adjacent to 8 provinces (Heilongjiang, Jilin, Liaoning, Hebei, Shanxi, Shaanxi, Ningxia and Gansu province), spans the Northeast, North and Northwest China, and shares a 4200 km border with Mongolia and Russia in the north (Figure 1).

Inner Mongolia has a vast territory and complicated landforms with the ecosystem structure composed by forest, grassland, desert and agro-pastoral zone. The unique ecosystem structure laid an important environmental foundation for the rich mineral resource and grain production in Inner Mongolia, forming a resource pattern of “forest in the east and mining in the west, farming in the south and grazing in the north”.

The scarce precipitation and the increasing demand for water resources due to the rapid economic development seriously affected the already fragile ecological environment system, which is mainly manifested as degradation of ecosystem and desertification in agricultural and pastoral zone, reduction in forest area and regional water shortage. The per capita water resource of Inner Mongolia is 2081 m^3^/person, which is in the middle range, and the average annual water modulus of Inner Mongolia is only 44,000 m^3^/km^2^, far lower than the national average level of 295,000 m^3^/km^2^.

Under the influence of natural geographical location and topography, there is a great disparity in water resources between the northeast and southwest [18]. There are five groundwater resources zones in Inner Mongolia (Figure 1), respectively, Songhua River Groundwater Zone (GW_I), Liao River Groundwater Zone (GW_II), Yellow River Groundwater Zone (GW_III), Hexi Corridor Groundwater Zone (GW_IV: extreme arid region) and Inner Mongolia High Plain Groundwater Zone (GW_V:).

GW_I and GW_II are in the temperate continental climate, with the annual precipitation of 274–619 mm and 248–514 mm in 2003–2021. The evaporation was 343–461 mm and 312–416 mm in 2003–2020 for GW_I and GW_II. The major land use is forest in GW_I, and cultivated land is concentrated in GW_II. Due to the large amount of water withdrawal, groundwater level decreases in GW_II, and water resources shortage is serious.

The annual precipitation is 155–407 mm in 2003–2021 for GW_III, and the annual evaporation was 238–359 mm in 2003–2020. Most of this area is grassland, the rest is cultivated land. Yellow River transit water and groundwater are the main water source for regional industrial and agricultural development and non-zonal grassland ecosystem.

The land use is bare land in GW_IV. GW_IV is dry and water-poor, with annual precipitation of 58–176 mm in 2003–2021 and annual evaporation of 78–119 mm in 2003–2020. There is a shortage in regional water resources, there are no perennial rivers and mainly groundwater is used.

The land use of GW_V is mainly the grassland, and the rest is cultivated land. The annual precipitation and evaporation in this region were 149–384 in 2003–2021 and 213–315 in 2003–2020, respectively.

### 2.2. Datasets

An overview of the datasets can be found in Table 1. A detailed description of these data is provided below.

(1) Gridded TWS anomalies (TWSA) data (relative to the 2004–2009 mean baseline in equivalent water height) were derived from the Gravity Recovery and Climate Experiment (GRACE) and GRACE Follow-On (GFO) mascon solution, provided by University of Texas Center for Space Research (CSR). More details about the CSR RL06 mascon (CSR-M) can be found in [58]. The spatial resolution was 1° × 1°, and temporal resolution was monthly. The GRACE was launched in March 2002 and collected over 15 years of time-variable gravity measurements prior to decommissioning in November 2017. GFO was launched in May 2018, and it obtained over four years of gravity observations to date. Cubic spline interpolation was used to fill in the missing data.
(2) Soil moisture (SM) and snow water equivalent depth (SWE) were derived from NOAH in Global Land Data Assimilation System (GLDAS), which ingested satellite- and ground-based observational data by land surface models and to generate the land surface states and fluxes [59]. The data spans, temporal resolution and spatial resolution were the same with GRACE mascon solution.
(3) The monthly gridded precipitation data was obtained from Global Precipitation Measurement (GPM) with spatial resolution of 0.1° × 0.1° [26]. These data were validated by cross-validation and error analysis with gauge-based precipitation. The monthly gridded evapotranspiration was provided by Global Land Evaporation Amsterdam Model (GLEAM) with a spatial resolution of 0.25° × 0.25° [29].
(4) The lake areas in Inner Mongolia were interpreted by satellite images from Landsat for 2000–2018 and Sentinel-2 for 2019–2021. Modified Normalized Difference Water Index (MNDWI) combined with Normalized Difference Vegetation Index (NDVI) and Enhance Vegetation Index (EVI) were calculated based on above satellite images to acquire information on lake areas. The water area and volume information of Hulun Lake were obtained from Hydroweb.
(5) Yellow River runoff data were obtained from Yellow River Water Resources Bulletin (Yellow River Conservancy Commission of the Ministry of Water Resources, 2003–2020).

### 2.3. Methods

#### 2.3.1. The GWS Changes Based on GRACE/GFO

Under the assumption that the changes of water stored in rivers, lakes and reservoirs are negligible, GWS anomalies could be calculated as the following equations:

(1) GWSA=TWSA−SMA−SWEA 

(2) $$GWSC=\frac{GWSA\left(t\right)-GWSA\left(t-1\right)}{t-\left(t-1\right)}\;$$

where *GWSA* is the GWS anomalies, *GWSC* is the GWS changes, *TWSA* is from GRACE/GFO, *SMA* and *SWEA* are the anomalies of SM and SWE from GLDAS. To unify the parameters, the SMA and SWEA calculated monthly data from the 2004–2009 mean value.

#### 2.3.2. Contribution of Different Hydrological Components to TWS Changes

The component contribution ratio (*CCR*) proposed by Kim et al. [60] was used to reflect the contributions of different hydrological components to TWS changes. The ratio was calculated as:

(3) $$CCR=\frac{MAD}{TV}\;$$

(4) $$MAD=\frac{\sum_{t}^{N}\left|S_{t}-\bar{S}\right|}{N}\;$$

where *MAD* is the mean absolute deviation of the hydrological component, *TV* is the total variability and is calculated as summation of all components’ MAD. $S_{t}$ is the value of component *S* (SWE, SM and GWS) at time *t* and N is the number of months, $\bar{S}$ is the average of $S_{t}$.

#### 2.3.3. Isolation the Natural and Human Induced TWS Changes

In order to quantify the human-induced TWS changes, the total TWS changes from GRACE/GFO and natural TWS changes based on climate water balance were compared.

The total TWS changes based on GRACE/GFO are expressed as Equation (5):

(5) $$TWSC=\frac{TWSA\left(t\right)-TWSA\left(t-1\right)}{t-\left(t-1\right)}\;$$

where *TWSA* is the TWS anomalies from GRACE/GFO.

The natural TWS changes are derived by water balance approach without considering the human activities. Natural TWS changes are deduced by monthly precipitation, evapotranspiration and surface runoff as in Equation (6):

(6) $$TWSC_{nat}=Pre-Evap-Runoff\;$$

where *Pre* is the precipitation from GPM, *Evap* is the evapotranspiration from GLEAM, and *Runoff* the surface runoff. As Inner Mongolia is located in an endorheic arid region, where the surface runoff is limited, the surface runoff was ignored in this method.

The TWS changes based on Equation (5) were combined with TWS changes based on Equation (6) to isolate the human-activities-induced TWS changes (*TWSC_hum_*).

(7) $$TWSC_{hum}=TWSC-TWSC_{nat}\;$$

#### 2.3.4. Statistics Analysis

The correlation analysis can reveal the relationship between two sequences. In this study, the Pearson correlation coefficient (*r*) was used to quantitative two sequences.

$$r=\frac{\sum_{i=1}^{n}\left(X_{i}-\bar{X}\right)\left(Y_{i}-\bar{Y}\right)}{\sqrt{\sum_{i=1}^{n}{\left(X_{i}-\bar{X}\right)}^{2}\sum_{i=1}^{n}{\left(Y_{i}-\bar{Y}\right)}^{2}}}\;$$

Root mean square error (*RMSE*) was used to evaluate the influences of surface water changes on local TWS\GWS changes.

$$RMSE=\sqrt{\frac{1}{n}\sum_{i=1}^{n}{\left(X_{i}-Y_{i}\right)}^{2}}$$

## 3. Results

### 3.1. The TWS Changes Based on GRACE/GFO

**Figure 2:**
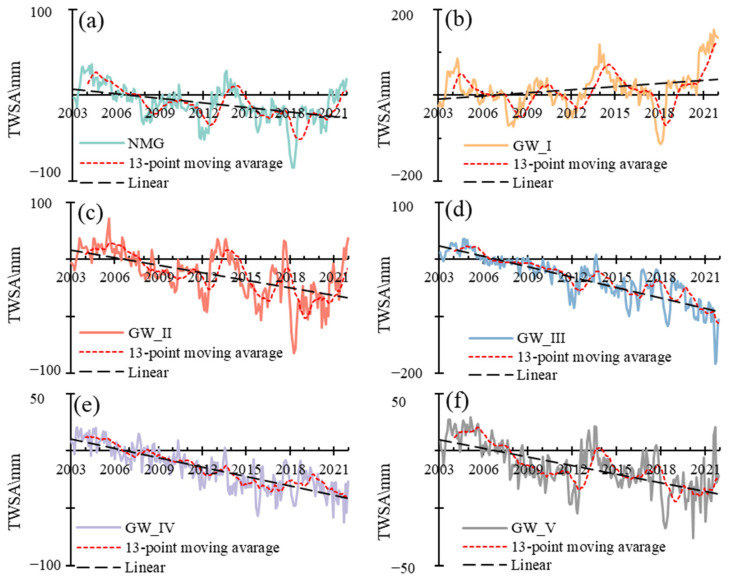
The temporal changes of TWSA in Inner Mongolia and different groundwater zones in 2003–2021. (**a**) The temporal changes of TWSA in Inner Mongolia in 2003–2021, (**b**) The temporal changes of TWSA in GW_I in 2003–2021, (**c**) The temporal changes of TWSA in GW_II in 2003–2021, (**d**) The temporal changes of TWSA in GW_III in 2003–2021, (**e**) The temporal changes of TWSA in GW_IV in 2003–2021, (**f**) The temporal changes of TWSA in GW_V in 2003–2021.

Figure 2 showed the TWSA variations in Inner Mongolia based on GRACE/GFO in the period of 2003–2021. The TWS varied obviously in different groundwater zones. The average TWSA was −25.43 mm, −33.97 mm, −15.83 mm and −13.95 mm for GW_II, GW_III, GW_IV and GW_V, respectively, indicating the deficit of land water storage. I average TWSA was 13.82 mm for GW_I, indicating the surplus land water storage.

The trends of TWS changes were also different for different groundwater zones (Figure 2). In GW_I, the TWS increased with the rate of 2.14 mm/a during 2003–2021, which significantly increased with the rate of 47.22 mm/a in 2018–2021 (Figure 2b). In GW_II, the TWS decreased with the rate of 4.62 mm/a during 2003–2021, decreased with the rate of 5.46 mm/a in 2003–2015 and increased with the rate of 28.69 mm/a in 2018–2021 (Figure 2c). The TWS decreased with the rate of 5.89 mm/a and 2.79 mm/a during 2003–2021 for GW_III and GW_IV (Figure 2d,e). In GW_V, the TWS decreased with the rate of 2.62 mm/a during 2003–2021, decreased with the rate of 5.80 mm/a in 2003–2011 and increased with the rate of 5.73 mm/a in 2018–2021 (Figure 2f).

### 3.2. The Surface Water Changes in Inner Mongolia

As the river water is limited in Inner Mongolia, this part mainly introduced the variation characteristics of lakes area based on satellite images and snow water based on GLDAS.

#### 3.2.1. The Changes of Lakes Area Based on Satellite Images

To better describe the changes of different-sized lakes, the lakes were categorized into three classes: small (<1 km^2^), medium (1–10 km^2^) and large (>10 km^2^). GW_I had the richest lake water resources, with a total of 107 lakes, of which small, medium and large lakes accounted for 33%, 55% and 12%, respectively. Hulun Lake, the largest lake in Inner Mongolia, was in GW_I with an area of more than 1500 km^2^. Influenced by Hulun Lake, the area decreased at the rate of 34.67 km^2^/a in 2003–2012, and increased at the rate of 24.17 km^2^/a in 2013–2021 for large lakes in GW_I.

A total of 74 lakes were detected across GW_II in 2003–2021, of which small, medium and large lakes accounted for 35%, 61% and 4%, respectively. The lake area was relatively stable for lakes in GW_II. The average areas for small, medium and large lakes were 12.18 km^2^, 115.70 km^2^ and 41.58 km^2^, respectively.

A total of 82 lakes were detected across GW_III in 2003–2021, of which small, medium and large lakes accounted for 20%, 76% and 5%, respectively. The lake area was also stable for lakes in GW_III, with average areas of 11.84 km^2^, 165.50 km^2^ and 203.96 km^2^ for small, medium and large lakes, respectively.

The lake water resources were limited in GW_IV. Only 28 lakes were detected across GW_IV in 2003–2021, 46%, 46% and 7% for small, medium and large lakes, respectively. The average areas were 6.20 km^2^, 32.03 km^2^ and 53.36 km^2^ for small, medium and large lakes, respectively. The lake area increased at the rate of 1.75 km^2^/a in 2003–2021.

**Figure 3:**
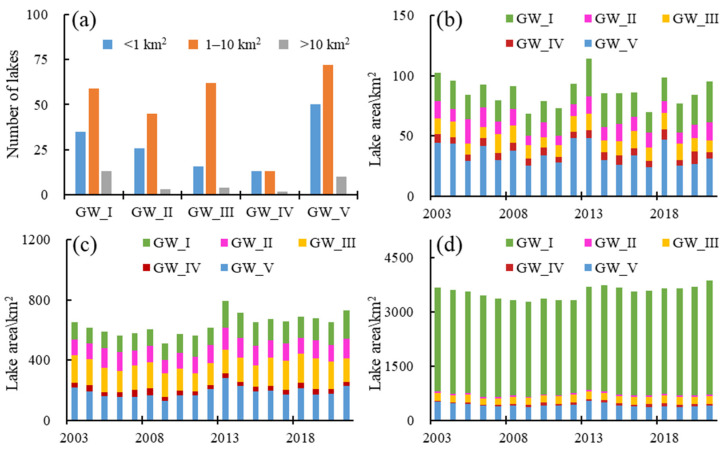
The number of lakes in different size (**a**), the temporal changes of lake area in small lakes (**b**), medium lakes (**c**) and large lakes (**d**) in 2003–2021.

A total of 132 lakes were detected in GW_V in 2003–2021, with 38%, 55% and 8% for small, medium and large lakes, respectively. The average total area was 645.32 km^2^, with 34.46 km^2^, 188.89 km^2^ and 421.98 km^2^ for small, medium and large lakes, respectively. The area decreased at the rate of 2.67 km^2^/a for lakes in GW_V in 2003–2021 (Figure 3).

#### 3.2.2. The Changes of Snow Water Based on NOAH

**Figure 4:**
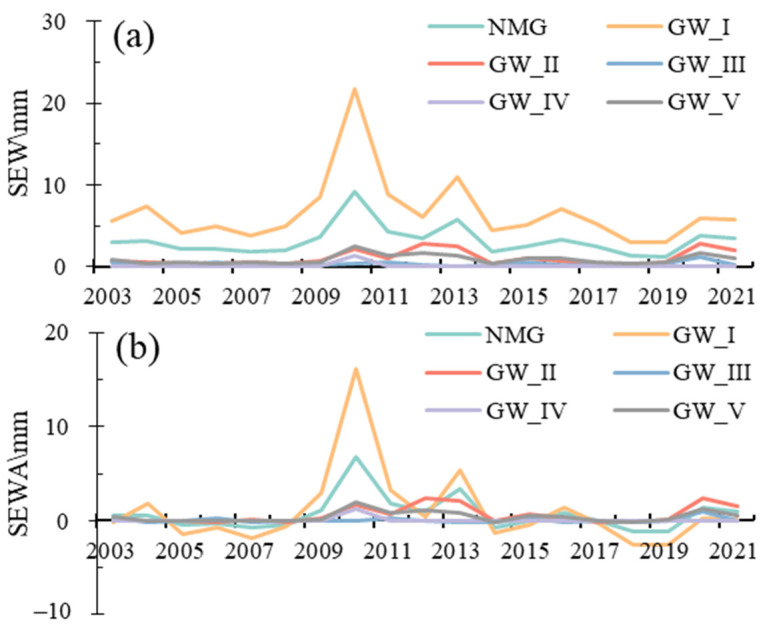
The temporal changes of snow water equivalent depth (SEW) (**a**) and snow water equivalent depth anomalies (SEWA) (**b**) in Inner Mongolia and different groundwater zones in 2003–2021.

The SWE from NOAH, GLDAS, was used to reveal the changes of snow water. There were significant differences in snow water among different regions. The average monthly\annual snow water equivalent depth (SWE) was 3.19\3.58 mm for Inner Mongolia. The average monthly\annual SWE was 6.67\7.38 mm, 1.06\1.34 mm, 0.35\0.36 mm, 0.13\0.15 mm and 0.91\1.07 mm in GW_I, GW_II, GW_III, GW_IV and GW_V, respectively (Figure 4a). The average monthly\annual snow water equivalent depth anomalies (SWEA) were 1.04\1.75 mm, 0.60\0.88 mm, 0.05\0.06 mm, 0.05\0.07 mm and 0.42\0.58 mm for GW_I, GW_II, GW_III, GW_IV and GW_V, respectively (Figure 4b).

### 3.3. The SM Changes Based on NOAH

**Figure 5:**
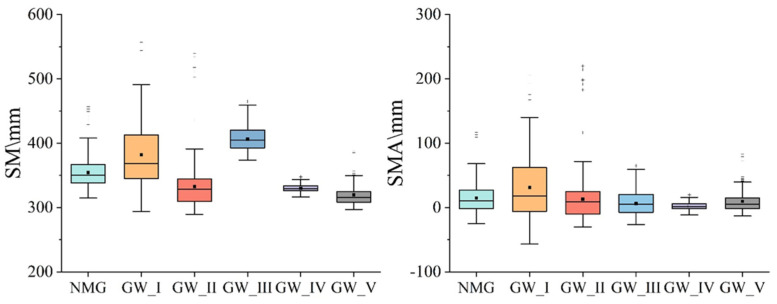
The box plot of soil moisture (SM) and soil moisture anomalies (SMA) in Inner Mongolia and different groundwater zones.

The average SM was 354.55 mm for Inner Mongolia in 2003–2021. The order of average SM was GW_III > GW_I > GW_II > GW_IV > GW_IV, with 382.10 mm, 332.72 mm, 406.34 mm 329.98 mm and 319.89 mm, respectively, while the average soil moisture anomalies (SMSA) were surplus for Inner Mongolia with the order of GW_I > GW_II > GW_V > GW_III > GW_IV (Figure 5).

**Figure 6:**
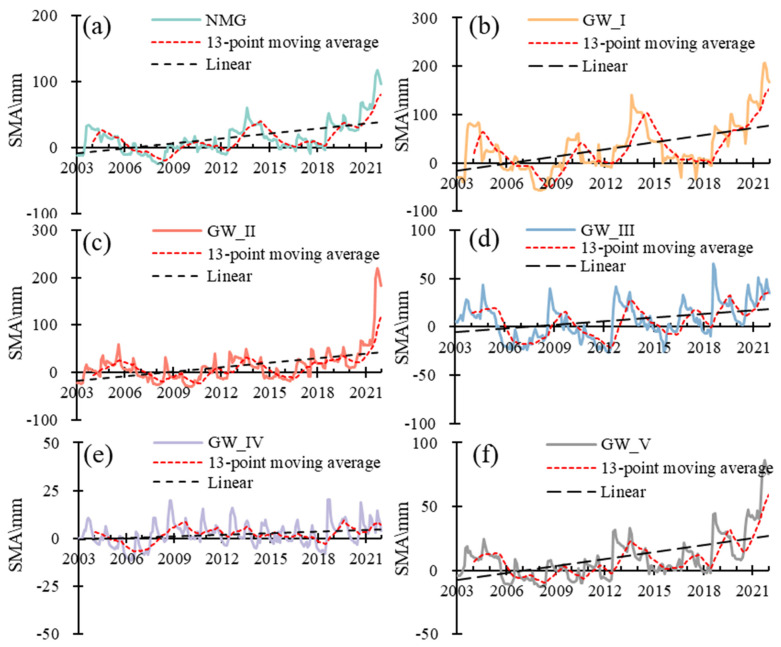
The temporal changes of soil moisture (SMSA) in Inner Mongolia and different groundwater zones in 2003–2021. (**a**) The temporal changes of SMSA in Inner Mongolia in 2003–2021, (**b**) The temporal changes of SMSA in GW_I in 2003–2021, (**c**) The temporal changes of SMSA in GW_II in 2003–2021, (**d**) The temporal changes of SMSA in GW_III in 2003–2021, (**e**) The temporal changes of SMSA in GW_IV in 2003–2021, (**f**) The temporal changes of SMSA in GW_V in 2003–2021.

In GW_I, the SMA increased with the rate of 4.17 mm/a from 2003 to 2021, with two increase stages and two decrease stages (Figure 6b). The SMA increased with a rate of 15.01 mm/a and 38.49 mm/a in 2008–2013 and 2018–2021 and decreased with a rate of 24.91 mm/a and 20.06 mm/a in 2004–2007 and 2014–2017, respectively. In GW_II, the SMA increased with the rate of 2.13 mm/a during 2003–2021 and 17.50 mm/a in 2018–2021 (Figure 6c). In GW_III, the SMA increased with the rate of 1.2 mm/a during 2003–2021 (Figure 6d). In GW_IV, the SMA was stable and only increased with the rate of 0.25 mm/a from 2003 to 2021 (Figure 6e). In GW_V, the SMA increased with the rate of 1.36 mm/a during 2003–2021 and 12.60 mm/a in 2018–2021 (Figure 6f).

### 3.4. The GWS Changes Based on Water Balance

**Figure 7:**
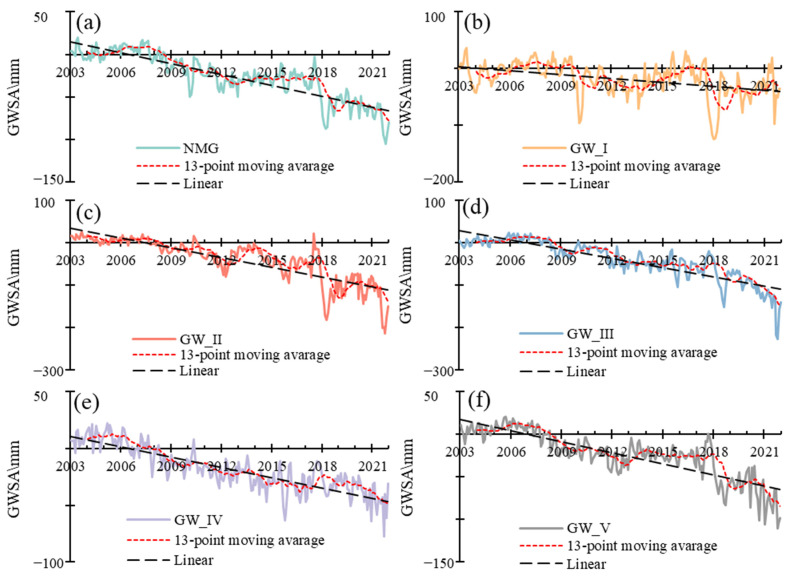
The temporal changes of GWSA in Inner Mongolia and different groundwater zones in 2003–2021. (**a**) The temporal changes of GWSA in Inner Mongolia in 2003–2021, (**b**) The temporal changes of GWSA in GW_I in 2003–2021, (**c**) The temporal changes of GWSA in GW_II in 2003–2021, (**d**) The temporal changes of GWSA in GW_III in 2003–2021, (**e**) The temporal changes of GWSA in GW_IV in 2003–2021, (**f**) The temporal changes of GWSA in GW_V in 2003–2021.

There were successful studies calculating the GWS change in arid regions based on GRACE\GFO data [55,61,62,63,64,65]. Like most studies, the GWS changes in Inner Mongolia were calculated. The GWS decreased with a rate of 2.21 mm/a, 6.76 mm/a, 6.87 mm/a, 3.01 mm/a and 4.14 mm/a for GW_I, GW_II, GW_III, GW_IV and GW_V, respectively, for 2003–2021 (Figure 7).

### 3.5. The Spatial Changes of Water Resources in Inner Mongolia

**Figure 8:**
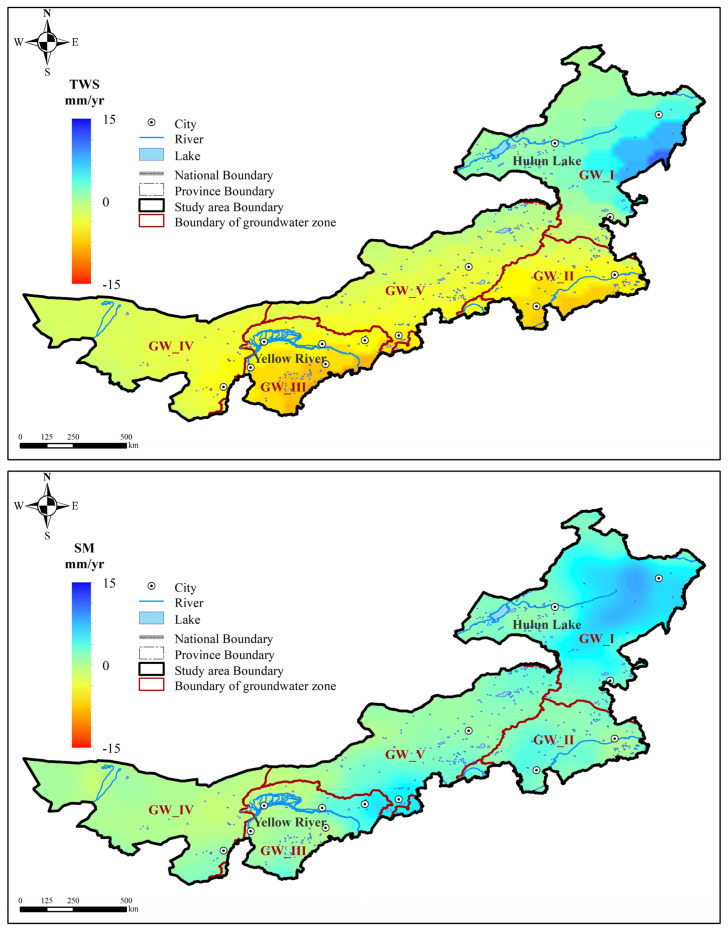

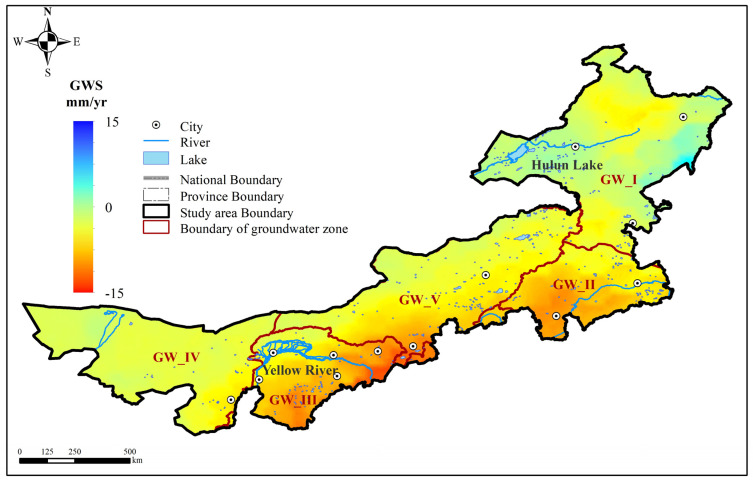
The spatial distribution of the change rate of TWS, SM, GWS in Inner Mongolia.

There were great spatial differences for water resources changes in Inner Mongolia. TWS changed at the rate of −9.59–11.39 mm/a, decreased in most regions and only increased in the northeast region. Soil moisture changed with small rate of −1.21–8.70 mm/a, increased in most of other regions and only decreased in the western area. Compared with TWS and soil moisture, the change rates of lake water and snow cover were small and could be ignored. Therefore, the GWS changed at the rate of −13.27–5.32 mm/a, almost decreased in whole Inner Mongolia and only increased in a small region of the northeast regions (Figure 8).

## 4. Discussion

### 4.1. Contribution of Hydrological Components to TWS Changes

Like most studies based on GRACE\GFO data, we decomposed the TWS changes into three key vertical water storage components: snow water, soil moisture and groundwater, and we ignored the changes in surface runoff and lake water due to the limited surface water resources in arid and semiarid regions. Understanding the contributions of above different hydrological components to the total TWS changes is crucial for investigating how the changes in individual component can potentially affect the availability and utilization of water resources.

Firstly, the influences of snow water, soil moisture and groundwater to TWS changes were qualitatively evaluated by correlation analysis. Table 2 showed the Pearson coefficients of TWS changes with snow water, soil moisture changes and groundwater storage changes. In GW_I, TWS changes showed high correlation with soil moisture changes (R^2^ = 0.91). In GW_II, TWS changes were positively correlated with both soil moisture changes and groundwater changes (R^2^ = 0.56 and R^2^ = 0.69, respectively). TWS changes showed high correlation with groundwater changes in GW_III, GW_IV and GW_V, and the coefficients were 0.66, 0.79 and 0.65, respectively.

**Figure 9:**
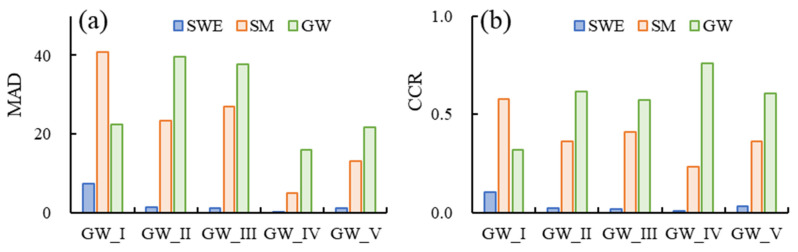
The mean absolute deviation (MAD) (**a**) and component contribution ratio (CCR) (**b**) in different groundwater regions in Inner Mongolia.

Secondly, the component contribution ratio (CCR) was used to quantitatively calculate the contributions of snow water, soil moisture and groundwater to TWS changes in different groundwater regions. Figure 9 showed the MAD and CCR in different groundwater regions based on Equations (3) and (4). The CCR indicated that the soil moisture was the major contributor to TWS changes in GW_I, followed by groundwater. Soil moisture and groundwater accounted for 58% and 32% of total TWS changes in GW_I, while groundwater was the major contributor to TWS changes in other four groundwater regions. The groundwater contributed 76% of total TWS changes in GW_IV, and soil moisture only accounted 23%. Groundwater contributed 62% and 57% of the total TWS changes in GW_II and GW_III, respectively, followed by soil moisture of 36% and 41%. In addition, changes in snow cover in the northeast also contributed to 10% of the total regional TWS changes.

**Figure 10:**
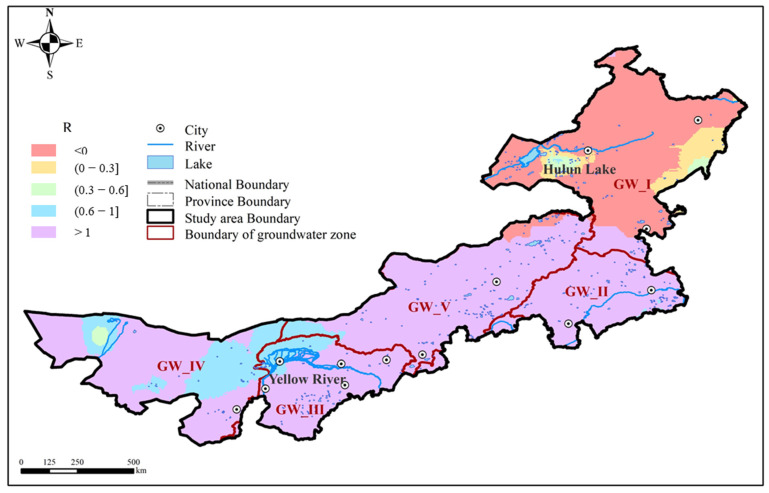
The spatial distribution of the ratio of GWS change rate with TWS change rate in Inner Mongolia.

In order to reveal the spatial distribution of contribution from groundwater to TWS changes, an index ^®^ was defined by the change rate of GWS changes with the rate of TWS changes. From Figure 10, there were five conditions for this index:

(1) The ratio was negative in the northeast region of Inner Mongolia. In this area, the TWS and soil moisture both showed an increase trend, while GWS showed a decrease trend. Soil moisture controlled the total TWS changes, and groundwater contributed negatively to the TWS changes.
(2) The ratio was 0–0.3 in a small part of GW_I. In this area, TWS, soil moisture and groundwater all increased. In this condition, soil moisture and groundwater both contributed positively to the changes of total water storage, but the soil moisture was also the major contributor.
(3) The ratio was between 0.3 and 0.6 in the area near Hulun Lake and Hei River. In these areas, TWS, soil moisture and groundwater all decreased, and soil moisture and groundwater contributed to the TWS changes in similar weight.
(4) The ratio was between 0.6 and 1 in the area near the Hei River and eastern of GW_IV. In these regions, TWS, soil moisture and groundwater all decreased, with groundwater decreasing at a faster rate. TWS changes were mainly controlled by groundwater.
(5) The ratio was higher than 1 in the rest areas, where the soil moisture increased and groundwater and TWS decreased. In these areas, groundwater was the major contributor of TWS changes.

### 4.2. The Role of Surface Runoff and Lake Water on Local Water Resources

Surface water is a key hydrological component, an important factor influencing the water cycle and ecological environment [66]. Kim, Yeh, Oki and Kanae [60] evaluated the role of a river in total TWS changes over global basins and concluded that neglecting river storage may lead to a mismatch in the amplitude and phase of TWS seasonal variations. In addition, studies showed that most of the lakes in semiarid and arid northern China experienced an obviously reduction in past decades [67,68,69]. Tao et al. [70] revealed that Inner Mongolia experienced significant lake shrinkage during the past several decades due to the unsustainable mining boom and agricultural irrigation, and the total water area of the lakes decreased from 4160.2 km^2^ in the late 1980s to 2900.6 km^2^ in 2010 with a decrease of 30.3%.

Despite this, most of the previous studies of GRACE/GFO hydrology applications did not consider the roles of surface water, and the impacts of surface runoff and lakes on TWS\GWS changes in arid regions did not receive enough attention relative to soil moisture and groundwater. Therefore, we raised a question that the lake water or surface runoff may have significance to the local water resources changes. To test this idea, we discussed the influences of Yellow River runoff changes and Hulun lake storage changes on local TWS\GWS changes in the respective groundwater resources zone.

#### 4.2.1. The Influence of Yellow River on TWS and GWS Changes in GW_III

Yellow River, the second longest river in China, flows through Inner Mongolia in GW_III. The runoff of Yellow River showed an increase trend from 2003 to 2021. The contribution of Yellow River runoff to the TWS in GW_III was calculated by the difference between the inflow and outflow of Yellow River. For consistency, the Yellow River runoff was processed as the GRACE/GFO data, i.e., anomalies were calculated relative to 2004–2009.

**Figure 11:**
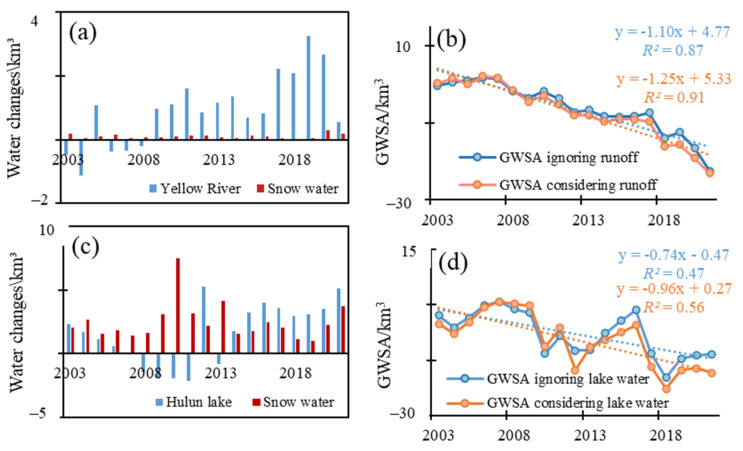
The changes of Yellow River runoff and snow water (**a**), the GWSA ignoring Yellow River runoff and considering the runoff in GW_III (**b**), the changes of Hulun Lake water volume and snow water (**c**) and GWSA ignoring Hulun lake water and considering Hulun lake water in GW_I (**d**).

The average Yellow River runoff anomalies in 2003–2021 was 0.94 km^3^, which was obviously higher than that of the snow water in GW_III (0.11 km^3^) (Figure 11a). This indicated that the Yellow River runoff had higher weight compared with the snow water. However, the Yellow River runoff was not enough to change the TWS trend in GW_III. The Yellow River runoff anomalies increased at a rate of 0.15 km^3^/a, while the annual TWS in GW_III decreased at a rate of −0.91 km^3^/a.

GWS changes in GW_III were underestimated if the runoff of Yellow River was not considered (Figure 11b). The annual average GWSA in GW_III was −6.19 km^3^ ignoring the Yellow River and −7.13 km^3^ considering the Yellow River. The root mean square error (RMSE) was 1.45 km^3^ between GWSA ignoring the runoff and GWSA considering the runoff. In addition, the GWS in GW_III decreased at a rate of 1.25 mm/a considering the runoff, which was higher than that which ignored the runoff (1.1 mm/a). Therefore, the runoff of Yellow River should not be neglected when analyzing the GWS changes in GW_III.

#### 4.2.2. The Influence of Hulun Lake on TWS and GWS Changes in GW_I

Hulun Lake, which is the largest lake in Inner Mongolia and the fifth largest lake in China, is located in GW_I. The water area and water level of Hulun Lake experienced obviously changed [71,72]. Based on the data from Hydroweb, the lake area decreased from more than 2000 km^2^ to around 1500 km^2^ in 1992–2012 and increased to more than 2100 km^2^ in 2022. The lake height decreased from around 545 m to 540 m in 1992–2012 and increased back to 545 m in 2022.

The lake water volume anomalies were calculated by abstracting the average value of 2004–2009. The average of lake water volume anomalies in 2003–2021 was 1.57 km^3^, less than that of snow water (2.48 km^3^) (Figure 11c). This was due to the rich snow resources in GW_I.

GWS changes were underestimated if the lake water volume was not considered (Figure 11d). The annual average GWSA was −7.85 km^3^ ignoring the lake water volume, and −9.43 km^3^ considering the lake water volume. The RMSE was 2.64 km^3^ between GWSA ignoring the lake water and GWSA considering the lake water. Therefore, Hulun Lake should not be neglected when analyzing the GWS changes in GW_I. The GWS considering the lake water in GW_I decreased at a higher rate (0.96 mm/a) than that ignoring the lake water (0.74 mm/a). Therefore, the runoff of Yellow River should not be neglected when analyzing the GWS changes in GW_III.

According to the above analyses, the surface water, which did not receive much attention in previous studies, is an important water storage component. Additionally, the influences of surface runoff and lake water to local GWS changes are not negligible, based on water balance theory.

### 4.3. Natural and Human Activities Induced TWS Changes

**Figure 12:**
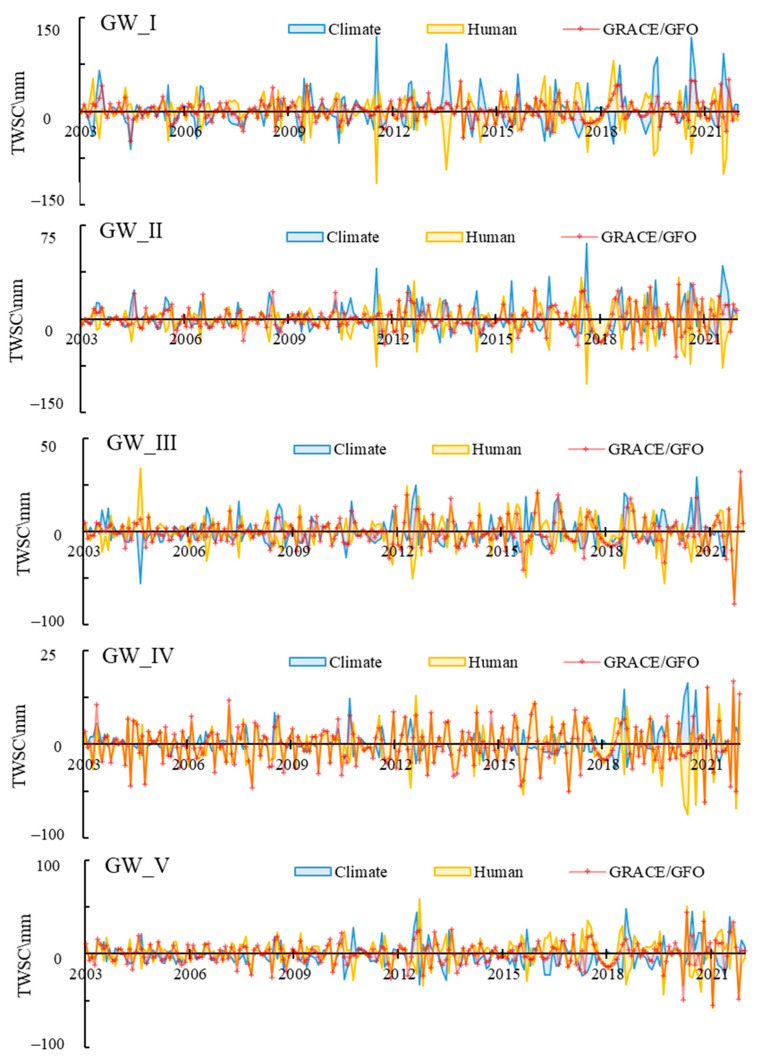
The monthly variations of natural-induced and human-induced TWS changes.

GRACE/GFO captures the total TWS changes caused by both natural and anthropogenic drivers. Natural TWS changes are estimated by climate water balance. Human-activities-induced TWS changes could be estimated by computing the difference between the total TWS changes and natural TWS changes. Figure 12 showed the comparison between monthly natural-induced, human-induced and integrated TWS changes.

As can be seen from Figure 12, a good agreement can be seen between total TWS changes and natural TWS changes in terms of the seasonal distribution for GW_I and GW_II. However, significant discrepancies were also apparent in terms of the magnitude. For example, the variation range of natural TWS in GW_I was −21.54–32.01 mm, which was greater than total TWS (−6.65–5.99 mm). This indicated that human activities significantly reduced the seasonality of TWS caused by meteorological factors, although TWS changes in GW_I tended to increase due to the natural precipitation replenishment.

For GW_III and GW_IV, there were obviously differences in the seasonality between total TWS changes and natural TWS changes. For example, the natural TWS showed the most surplus in September, but TWS changes based on GRACE/GFO was deficit in September. This indicated human activities changed the seasonality of TWS changes in these regions.

It was interesting that the natural TWS showed more serious losses than GRACE/GFO-based TWS in GW_V, −17.62 mm, and −1.01 mm, respectively. In spring (March –May), both natural TWS changes and total TWS changes were deficit, −27.47 mm, and −10.10 mm, respectively. In summer (June–August), both natural TWS changes and total TWS changes were surplus, 5.67 mm and 18.61 mm, respectively. The above phenomena indicated that human activities played a positive role in total TWS changes in this region in spring and summer. The average natural TWS change was 0.30 mm in autumn, while the total TWS change was −11.72 mm, indicating human activities played a negative role in total TWS changes. The average natural TWS changes and total TWS changes were similar in winter, which indicated that natural climate factors dominated the TWS of this region.

**Figure 13:**
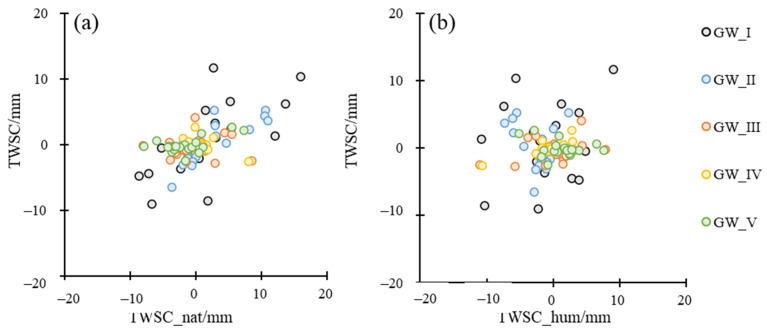
The relationship between natural TWS changes (TWSC_nat) with total TWS changes from GRACE/GFO data (TWSC) (**a**) and human-induced TWS changes (TWSC_hum) with TWSC (**b**).

Figure 13 compared the total TWS changes with natural TWS changes and human-activities-induced TWS changes in annual scale. Table 3 showed the correlation coefficient (R2) and RMSE between total TWS changes with natural TWS changes and human-activities-induced TWS changes in annual scale. Based on the above results, the total TWS changes were dominated by climate factors for GW_I and GW_II. Human activities explained the major TWS changes in IV. For the GW_III and GW_V, the situations were more complicated, it was hard to disentangle the contribution from climate factors and human activities.

Some studies showed that the changes of land water storage changes based on climate water balance were highly consistent with that based on GRACE [73,74]. However, the situation was different in this study, and this was not surprising for the discernable differences. We speculated these differences may be induced by the large water consumption caused by human-associated activities would overestimate the natural TWS changes. Without enough surface water, Inner Mongolia has little choice but to increase the reliance on groundwater to replenish the water for agricultural irrigation and coal mining [39]. Additionally, water storage changes in Inner Mongolia during the study period followed the typical pattern of increased groundwater abstraction in arid and semiarid regions [75]. Therefore, the deterioration of groundwater storage is expected to continue in the following decades not only because of the change climate but also due to increasing exploitation.

## 5. Conclusions

This study revealed the temporal and spatial changes of total TWS and their individual components in Inner Mongolia, China, using multiple remote sensing data for the period of 2003–2021, evaluated the contribution of hydrological components to TWS changes and isolated the human-induced TWS from total TWS changes.

Soil moisture and groundwater are the major contributors of TWS changes in Inner Mongolia. To be specific, soil moisture primarily contributes to TWS changes in Songhua River Groundwater Zone (GW_I), soil moisture and groundwater both contribute to Liao River Groundwater Zone (GW_II), and groundwater mainly composes the TWS changes in Yellow River Groundwater Zone (GW_III), Hexi Corridor Groundwater Zone (GW_IV) and Inner Mongolia High Plain Groundwater Zone (GW_V).

The contributions of surface runoff and lake water to TWS changes are usually ignored in arid and semi-arid areas. However, our analyses showed the runoff and lake water cannot be considered as an invariant constant which cannot change the major result about the groundwater changes.

In relation to natural- and human-induced changes in the terrestrial water cycle, the natural climate condition controls the water resources changes in GW_I and GW_II, while human activities influenced the water resources changes in GW_IV. Human activities changed the seasonality of TWS changes in GW_III and GW_V.

Due to the lack of in situ data available for validation, it is crucial to recognize that the analyses and resulting conclusions were subject to inherent uncertainty. However, we believe that this combination of multiple remote sense data provides a very valuable alternative for understanding hydrologic changes occurring in data-scarce regions.

## Figures and Tables

**Table 1:** Information of datasets used in this study.

Variables	Data	Period	Spatial Resolution	Temporal Resolution	Source
TWS change	GRACE/GFO	2003–2021	1° × 1°	monthly	http://www2.csr.utexas.edu (accessed on 13 October 2022)
Soil moisture	GLDAS	2003–2021	1° × 1°	monthly	https://disc.gsfc.nasa.gov/ (accessed on 13 October 2022)
Snow water					
Precipitation	GPM	2000–2021	0.1° × 0.1°	30 min	https://gpm.com.hk/ (accessed on 13 October 2022)
	gauges	2000–2020	-	monthly	China Meteorological Data Service Center (accessed on 13 October 2022)
Evaporation	GLEAM	2003–2021	0.25° × 0.25°	daily	http://www.gleam.eu (accessed on 13 October 2022)
Lake area	Landsat 8	2000–2018	30 m	monthly	https://glovis.usgs.gov/ (accessed on 13 October 2022)
	Sentinel 2	2019–2021	30 m	monthly	https://scihub.copernicus.eu/dhus/#/home (accessed on 13 October 2022)
	Hydroweb		-	daily	http://hydroweb.theia-land.fr (accessed on 13 October 2022)
Runoff data	Yellow River		-	annual	Yellow River Conservancy Commission of the Ministry of Water Resources (accessed on 13 October 2022)

**Table 2:** The Pearson coefficient of TWS changes with its hydrological components.

**GW_I**	**TWS**	**SMS**	**SWS**	**GWS**
TWS	1	0.906 **	0.382	0.183
SMS		1	0.436	−0.226
SWS			1	−0.419
GWS				1
**GW_II**	**TWS**	**SMS**	**SWS**	**GWS**
TWS	1	0.556 *	0.418	0.686 **
SMS		1	0.438	−0.222
SWS			1	0.050
GWS				1
**GW_III**	**TWS**	**SMS**	**SWS**	**GWS**
TWS	1	0.399	−0.377	0.660 **
SMS		1	−0.412	−0.426
SWS			1	−0.061
GWS				1
**GW_IV**	**TWS**	**SMS**	**SWS**	**GWS**
TWS	1	0.291	0.331	0.788 **
SMS		1	−0.046	−0.352
SWS			1	0.266
GWS				1
**GW_V**	**TWS**	**SMS**	**SWS**	**GWS**
TWS	1	0.345	0.333	0.649 **
SMS		1	0.229	−0.483 *
SWS			1	0.027
GWS				1

Notes: ** indicates the confidence coefficient is 0.01, and * indicates the confidence coefficient is 0.5.

**Table 3:** The correlation coefficient (*R*) and *RMSE* between total TWS changes with natural TWS changes and human-activities-induced TWS changes in annual scale.

	GW_I		GW_II		GW_III		GW_IV		GW_V	
	N	H	N	H	N	H	N	H	N	H
*R*	0.83	0.45	0.91	−0.50	0.28	0.59	−0.74	0.88	0.76	−0.53
*RMSE*	5.05	6.82	3.39	5.08	3.98	3.76	2.85	2.18	3.32	3.83

## Data Availability

The GRACE and GRACE Follow-On data were collected from http://www2.csr.utexas.edu/GRACE/RL06_mascons.html (accessed on 13 October 2022). The GLDAS data were collected from http://disc.sci.gsfc.nasa.gov/serVICes/grads-gds/gldas/, accessed on 13 October 2022). The gridded precipitation data were obtained from Global Precipitation Measurement (GPM) (https://gpm.com.hk/, accessed on 13 October 2022). The evapotranspiration data were from Global Land Evaporation Amsterdam Model (http://www.gleam.eu, accessed on 13 October 2022).
